# Assessment of the measurement properties of the Brazilian versions of the Functional Status Score for the ICU and the Functional Independence Measure in critically ill patients in the intensive care unit

**DOI:** 10.5935/0103-507X.20190065

**Published:** 2019

**Authors:** Giovani Assunção de Azevedo Alves, Bruno Prata Martinez, Adriana Claudia Lunardi

**Affiliations:** 1 Programa de Mestrado e Doutorado em Fisioterapia, Universidade Cidade de São Paulo - São Paulo (SP), Brasil.; 2 Hospital Aliança - Salvador (BA), Brasil.; 3 Departamento de Fisioterapia, Universidade do Estado da Bahia - Salvador (BA), Brasil.; 4 Departamento de Fisioterapia, Faculdade de Medicina, Universidade de São Paulo - São Paulo (SP), Brasil.

**Keywords:** Functional scales, Measurement property, Critical care, Activities of daily living

## Abstract

**Objective:**

To compare the measurement properties (internal consistency, intra and interrater reliability, construct validity, and ceiling and floor effects) of the Functional Status Score for the ICU (FSS-ICU) and the Functional Independence Measure (FIM-motor domain).

**Methods:**

In this study of measurement properties, the FSS-ICU and FIM were applied to 100 patients (72.1 ± 15.9 years; 53% male; Sequential Organ Failure Assessment = 11.0 ± 3.5 points, Simplified Acute Physiology Score 3 = 50.2 ± 16.8 points) in an intensive care unit at baseline and after 2 hours by physiotherapist 1 (test and retest) and 30 minutes after baseline by physiotherapist 2. The measurement properties evaluated were internal consistency (Cronbach's alpha), intra- and interrater reliability (intraclass correlation coefficient), agreement (standard error of measurement) and minimum detectable change at a 90% confidence level, ceiling and floor effects (frequency of maximum and minimum scores) and construct validity (Pearson's correlation).

**Results:**

The FSS-ICU and FIM presented adequate internal consistency (Cronbach's alpha, FSS-ICU = 0.95 and FIM = 0.86), intra-and interrater reliability for overall FSS-ICU and FIM score (ICC > 0.75), agreement (minimum detectable change at a 90% confidence level: FSS-ICU and FIM = 1.0 point; standard error of measurement: FSS-ICU = 2% and FIM = 1%) and construct validity (r = 0.94; p < 0.001). However, the FSS-ICU and FIM presented ceiling effects (maximum score for 16% of patients for the FSS-ICU and 18% for the FIM).

**Conclusion:**

The FSS-ICU and FIM present adequate measurement properties to assess functionality in critically ill patients, although they present ceiling effects.

## INTRODUCTION

Approximately 20 million people are admitted every year to intensive care units (ICU) around the world.^(1)^ Improvements in ICU care have allowed for increased survival of these patients; however, the impact on functionality has been an increasingly reported adverse effect.^(2-4)^

Functionality is conceptualized as the ability to perform activities ranging from self-care to activities that demand high strength and mobility.^(5)^ Some factors are associated with impaired functionality, where severity of the critical illness, use of sedatives and/or replacement therapies such as hemodialysis, need for invasive mechanical ventilation and prolonged bed rest are associated with impaired physical fitness and reduced functionality.^(1,2,6,7)^ This negative impact on physical fitness and functionality acquired in the ICU may compromise the patients' return to full social functioning for up to 5 years.^(8)^

In an attempt to minimize or prevent these adverse effects of ICU admission, early mobilization has been increasingly studied and implemented,^(2)^ with positive effects on the improvement of muscle strength, gait independence, reduced length of hospital stay and continuous improvements in functionality.^(1,9)^ However, to evaluate the impact of early mobilization in the ICU, it is essential to use instruments validated and tested in this setting.^(10)^ The first scales for functional evaluation in the ICU were adapted from rehabilitation centers to this setting and include the Functional Independence Measure - FIM.^(10,11)^ To use the FIM for the ICU setting, some modifications were necessary in the scale, such as the reduction of 18 evaluation items to only 2, with an emphasis on the most relevant tasks in the ICU setting.^(12-15)^

To improve the functional assessment process, functional scales were specifically developed for the ICU setting.^(16)^ Such scales include the Functional Status Scale for the ICU (FSS-ICU), which evaluates the ability to roll in bed, transfer from laying to sitting, transfer from sitting to standing, sit at the edge of the bed and walk.^(17-19)^ Higher final scores on the FIM and FSS-ICU indicate better functionality, and lower scores indicate dependency of the patients for the activities evaluated.^(20,21)^ The FIM and the FSS-ICU have already been validated and translated into Portuguese, but only the sensitivity of the FIM and the reliability of the FSS-ICU were previously tested.^(21,22)^ However, the assessment of reliability, ceiling and floor effects, and construct validity are part of a set of measurement properties that should be tested in health instruments to assist in selecting the most appropriate instrument for application in clinical practice.^(11,21,23,24)^

The objective of this study was to evaluate the measurement properties (convergent validity, intra- and interrater reliability, internal consistency, agreement, and ceiling and floor effects) of the FSS-ICU and of the motor domain of the FIM in patients admitted to the ICU.

## METHODS

This was a study of measurement properties, approved by the Human Research Ethics Committee of the *Universidade Cidade de São Paulo* under number 45685215.8.0000.0064.

All eligible patients signed an informed consent form. The patients evaluated were admitted consecutively to the ICU of the *Hospital Aliança* of Salvador, Bahia, Brazil, for clinical or surgical reasons, between January 2016 and December 2017. This facility is a private and medium-size hospital, with a general ICU with 15 beds, that treats clinical and surgical patients. Physiotherapists, physicians, nurses, nursing technicians, nutritionists, social workers and psychologists are part of the ICU team.

According to the guidelines of the Consensus-based standards for the selection of health measurement instruments (COSMIN), a sample of 100 patients is required to analyze reproducibility, construct validity and ceiling and floor effects. Therefore, a sample size of 100 patients was determined.^(25)^ The patients included were those admitted for more than 48 hours in the ICU, without clinical restrictions of mobilization, who were awake, cooperative and understood the requested commands and who agreed to participate in the study.

Once a patient was selected for the study, a consent form was presented to him/her and/or a family member during visiting hours. The scales were not applied the first time the patients got up from bed after long periods of immobilization due to the greater likelihood of orthostatic hypotension and the consequent fear of patients in performing the tests, which could compromise the reliability analyses. Mobilization after analgesia was also defined, in consultation with the nursing team, for those patients who required it.

Patients with an altered clinical status, detected by the medical team, between assessments (hypotension or hypertension, bradycardia or tachycardia, tachypnea and/or sensory changes relative to baseline), under the effect of sedation and vasoactive drugs and using invasive mechanical ventilation at the time of assessment were excluded.

All patients evaluated were prescribed relative rest confirmed by the attending physician and the stability of the physiological systems (respiratory, cardiovascular and mental); thus, the physiotherapist was able to evaluate the neuromusculoskeletal system by means of the FSS-ICU and the FIM-motor domain.

**Figure 1:**
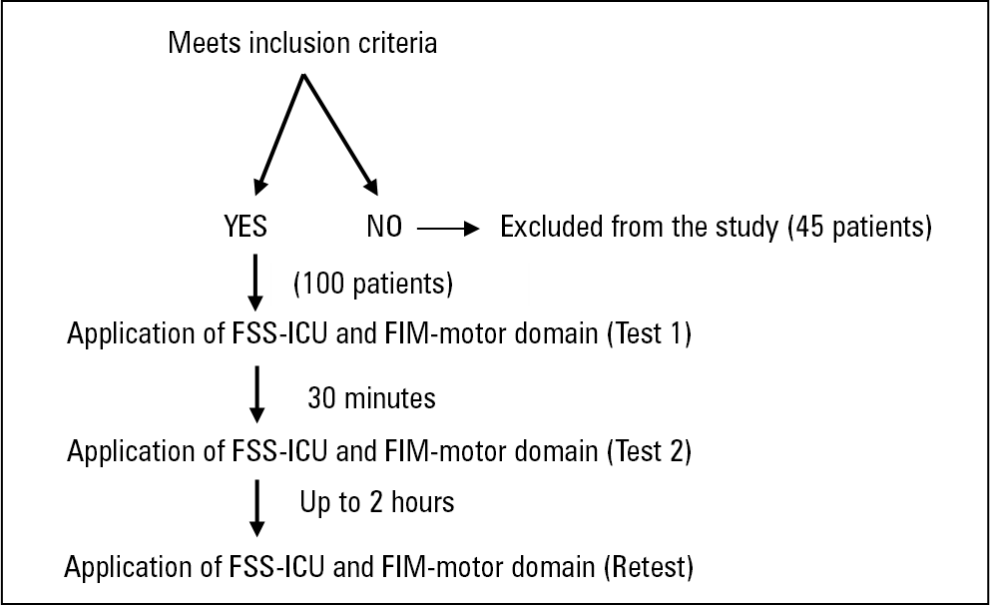
Flowchart for the application of the Functional Status Score - ICU and Functional Independence Measure - motor domain. FSS-ICU - Functional Status Score for the ICU; FIM - Functional Independence Measure.

To evaluate the patients, 15 physiotherapists were included in the study. All were intensive care specialists and trained on the application of the scales, which are routinely used in the hospital. Each patient was evaluated by 2 of these physiotherapists. Physiotherapist 1 began the procedures for assessment at baseline, applying the FSS-ICU and the FIM-motor domain (test 1) to analyze the internal consistency and the ceiling and floor effects. After 30 minutes, physiotherapist 2, blind to the results of test 1, also applied the FSS-ICU and the FIM-motor domain (test 2) to analyze interrater reliability. After 2 hours, physiotherapist 1 reapplied the scales (retest) for analysis of intrarater reliability and agreement (Figure 1). When recording the results on the data sheet, the physiotherapists used an instruction manual that details the levels of assistance used in the assessment.^(21)^

Age, sex, dates of ICU admission and discharge, reason for ICU admission, comorbidities, need for invasive mechanical ventilation with recording of intubation and extubation dates, and use of vasoactive drugs and sedatives were recorded in the data collection form. The Sequential Organ Failure Assessment (SOFA)^(26)^ and the Simplified Acute Physiology Score 3 (SAPS 3)^(27)^ were used to determine the clinical severity of patients.

The FSS-ICU is used to grade a patient's physical performance and evaluates 5 tasks: rolling in bed, transferring from laying to sitting, transferring from sitting to standing, sitting on the edge of the bed and walking. Each item can receive up to 7 points, depending on the degree of assistance required for each task. In total, the score can range from zero (totally dependent) to 35 (completely independent).^(21,28)^

The FIM-motor domain contains only the transfers and locomotion domains because we consider them to be the most relevant in the ICU for use by the physiotherapist and are also more comparable to the FSS-ICU.^(12)^ Its score ranges from zero to 7 for each task, considering the degree of assistance required for the patient to perform bed transfers and locomotion. The sum of the items ranges from a minimum value of zero to a maximum of 14 points.^(11,14)^

The tested properties and respective analyses are presented as the means and standard deviation for continuous variables and as absolute numbers, percentages and frequencies for the categorical variables, after applying the Shapiro-Wilks normality test.

The internal consistency was determined using Cronbach's alpha for the total FSS-ICU and FIM-motor domain scales. In addition to the total analysis, internal consistency without each of the items was also evaluated for the FSS-ICU for detecting redundancy. This index ranges from zero to 1, and the higher the value is, the greater the reliability of the scale. Values between 0.75 and 0.95 are considered appropriate.^(29)^

The agreement was tested by the standard error of measurement (SEM) and the minimum detectable change at a 90% confidence level (MDC_90_). The MDC_90_ corresponded to the score in test 1 minus the score in test 2 divided by the √2 × SEM. The SEM was considered very good if <5% of the total score, good if ≥ 5% and < 10%, doubtful if ≥ 10% and < 20%, and negative if > 20%.^(29)^

The intra- and interrater reliabilities were tested using the intraclass correlation coefficient (ICC), subtype absolute agreement for single measures, considering the variance in the measurements for each participant, not the mean (ICC_2.1_), with its respective 95% confidence interval (95%CI). The result was classified as follows: poor if ICC < 0.4; satisfactory if 0.4 ≤ ICC < 0.75 and excellent if ICC ≥ 0.75.^(29)^

The convergent validity was tested using Pearson's correlation between the FSS-ICU and the FIM-motor domain total scores. The correlation level was characterized as follows: *ƿ* = 1 indicated a perfect positive correlation between 2 categories of the scales; *ƿ* = -1 indicated a perfect negative correlation between 2 categories of the scales; and *ƿ =* 0 indicated that the categories of the scales were not linearly dependent on each other. Our hypothesis was that there was a strong and positive correlation between the scales because they evaluated the same construct (mobility).^(29)^

Ceiling and floor effects were tested by frequencies and were considered present if 15% or more patients reached a maximum or minimum score for the instruments.^(29)^

## RESULTS

Initially, 145 patients were screened, and 45 were excluded due to clinical changes between assessments (Figure 1). A sample of 100 patients with a mean age of 72.1 ± 15.9 years completed the study. Approximately 19% had 2 or 3 reasons for ICU admission, 70% of the sample had more than 1 associated comorbidity, and postoperative (elective or urgent) conditions were the main causes of ICU admission. The mean SOFA severity score was 11.0 ± 3.5, and the mean SAPS 3 score was 50.2 ± 16.5 (Table 1). For the patients who required it, the average time of invasive mechanical ventilation was 5.36 ± 4.34 days. Sedation was used for 26.9% of patients, and 28.8% required vasoactive drugs.

**Table 1:** Demographic and clinical characteristics of the patients

Variables	Mean ± SD	%
Age (years)	72.1 ± 15.9	
Male sex		53
Comorbidities		
Systemic arterial hypertension		62
Diabetes mellitus		31
Chronic renal failure		26
Cancer		25
Chronic obstructive pulmonary disease		24
Other		47
None		6
Causes of ICU admission		
Postoperative		31
Acute respiratory failure		25
Sepsis		21
Pneumonia		14
Other		15
Severity scores		
SOFA	11.0 ± 3.5	
SAPS 3	50.2 ± 16.5	
Use of invasive mechanical ventilation		34

SD - standard deviation; ICU - intensive care unit; SOFA - Sequential Organ Failure Assessment; SAPS 3 - Simplified Acute Physiology Score 3.

### Internal consistency

The internal consistency was considered adequate; Cronbach's alpha was 0.95 for the total FSS-ICU score (Table 2), and a Cronbach's alpha of 0.86 was obtained for the total FIM-motor domain score. It was not possible to calculate the Cronbach's alpha for each deleted item of the FIM-motor domain scale because it only has 2 items.

**Table 2:** Cronbach's alpha per deleted item for each task of the Functional Status Score - ICU

Activity	Cronbach's alpha
Rolling in bed	0.94
Lie-to-sit transfer	0.94
Sitting on the edge of the bed	0.95
Sit-to-stand transfer	0.93
Walking	0.95

### Agreement

The MDC_90_ was 1.0 point for the FSS-ICU and FIM, with SEMs of 0.54 points (2%) and 0.11 points (1%), respectively.

### Reliability

The ICCs of the total FSS-ICU score and total FIM-motor score were considered adequate and are provided in table 3.

**Table 3:** Intra- and interrater intraclass correlation coefficients for total Functional Status Score - ICU and Functional Independence Measure - motor domain

	Intrarater reliability (test and retest)	Confidence interval
FSS-ICU	ICC = 0.987 (95%CI 0.981 - 0.991)	ICC = 0.957 (95%CI 0.937 - 0.971)
FIM-motor domain	ICC = 0.955 (95%CI 0.934 - 0.969)	ICC = 0.953 (95%CI 0.931 - 0.968)

FSS-ICU - Functional Status Score for the ICU; ICC - intraclass correlation coefficient; 95%CI - 95% confidence interval; FIM - Functional Independence Measure.

### Convergent validity

A correlation of 0.94 was identified between the total FSS-ICU score and the total FIM-motor domain score, with p < 0.001.

### Ceiling and floor effects

The ceiling effect was observed in 16% of the sample for the FSS-ICU, and in 18% of the sample for the FIM. The floor effect was not observed in the scales. The mean total score for the FSS-ICU was 21.37 ± 10.07, and the mean total score for the FIM-motor domain was 8.06 ± 4.31.

## DISCUSSION

This study aimed to evaluate the measurement properties of the FSS-ICU and FIM-motor domain, and a strong correlation between them was identified because they evaluate the same construct: mobility. The results of this study demonstrated that the FSS-ICU and the FIM-motor domain showed adequate internal consistency, reliability and agreement for all tasks in the test and retest. Furthermore, the validity was convergent between the scales, and both had no floor effects but had ceiling effects.^(29)^

Based on the detection of adequate measurement properties in both scales, we can infer that although the FSS-ICU was developed using the FIM as a reference, it is a more complete and robust scale, being specific for the ICU setting, and allows documenting and measuring the efficacy of therapeutic interventions during the provision of care to critically ill in-hospital patients.^(17,19)^ Finally, the motor domain of the FIM has only 2 items, and its application is therefore quick and may be a good choice for screening and referring patients at functional risk for more or less intense physical therapy during ICU treatment. In addition, a 2-point change in the FSS-ICU seems to be clinically important in chronic patients, and the application of the FSS-ICU does not significantly alter the physiotherapy assessment or treatment sessions, as it is estimated that less than 1 minute is necessary to record the data and, therefore, it is important for documenting the effects of the interventions.^(19)^

As FIM was not developed for the hospital setting, a multicenter American study had already investigated the validation of this scale in that setting, where it was applied in subacute and chronic patients predominantly affected by neurological diseases. The study identified adequate internal consistency, although it was lower than 0.70 for the subitem locomotion, being lower than the value of 0.86 found in our study, in which the scale was applied to critically ill patients.^(30)^ Dodds et al.^(30)^ reinforced that the FIM is an instrument with adequate reliability for identifying the physical changes of patients in a rehabilitation program and confirmed the ability (construct validity) of the instrument to discriminate patients based on age and comorbidities.

The FIM was only translated and validated in Brazilian Portuguese in 2000, and the authors reported no linguistic and cultural equivalence issues, with convergent validity confirmed when applied to patients with greater motor impairment, who presented lower total scores.^(11)^ However, again, the reliability of the scale was lower in the study by Riberto et al.,^(11)^ which involved ICU patients and outpatients in the same sample when compared with our study exclusively with critically ill patients. Another difference between the studies was the use of the qualitative and quantitative scores to evaluate agreement. Riberto et al.^(11,14)^ evaluated the reliability of 2 evaluators using the kappa test and considered it to be moderate-substantial, unlike our study, which measured reliability using the ICC and scored it as excellent.

In 2010, the alpha FIM version reduced the scale from 18 items to 6 items (4 motor and 2 cognitive) and maintained a score of 1 to 7 to classify the level of independence for each task.^(13)^ This scale has already been tested in the ICU and was able to identify changes in the ability to perform transfers and locomotion between hospital admission and discharge.^(13)^ A previous study applied the FIM only with the bed transfer and locomotion domains in the ICU, using a score from 1 to 7 to classify the independence of patients to perform the tasks,^(12)^ confirming that this scale can be better applied in the ICU with these adaptations; however, the measurement properties of this change in scale were tested for the first time in our study.^(12)^

Unlike the FIM, the FSS-ICU was developed specifically for the ICU setting when it evaluated the functional impact in patients who required invasive mechanical ventilation,^(17)^ and its clinical usefulness was confirmed for the functional evaluation in a population of patients with long hospital stays.^(19)^ Table 4 shows the measurement properties of studies that used the FSS-ICU and FIM.

**Table 4:** Distribution of the measurement properties of the Functional State Scale - ICU and Functional Independence Measure

Author	Scale	Translation into Portuguese	Validity	Internal consistency	Concordance	Reliability	Ceiling and floor effects
Riberto et al.^(8)^	FIM	Yes	Identified functional gains				
Hinkle et al.^(10)^	Alpha FIM			Cronbach's alpha 0.90		Excellent ICC = 0,92	
Riberto et al.^(11)^	FIM	Yes	Convergent validity Association between higher motor impairment and lower scores		MDC_90%_ and SEM < 5%	Kappa Inter r = 0.87 (ICC = 0.87 - 0.98) Intra r = 0.91 (ICC = 0.91 - 0.98) Cronbach's alpha = 0.94 Test and retest = ICC > 0.95	
Silva et al.^(17)^	FSS-ICU	Yes				Interrater Rolling = 0.84 (95%CI = 0.54 - 0.94) Lie-to-sit transfer = 0.86 (95%CI = 0.68 - 0.94) Sit-to-stand transfer = 0.85 (95%CI = 0.57 - 0.94) Sitting at edge of the bed = 0.90 (95%CI = 0.77 - 0.96) Walking = 0.91 (95%CI = 0.80 - 0.94) Total FSS-ICU score = 0.88 (95%CI = 0. 73 - 0.95)	
Huang et al.^(24)^	FSS-ICU		High scores associated with reduced length of stay	Cronbach's alpha 0.78 – 0.95	SEM: 1.8 MDC_90%_: 2.4 - 4.1		Floor: 0.5% Ceiling: 21%
Dodds et al.^(26)^	FIM		Low scores associated with older age and more comorbidities	Cronbach's alpha Admission: 0.93 Discharge: 0.95			
Ragavan et al.^(33)^	FSS-ICU					Intrarater: ICC = 0.98 (95% CI = 0.96 - 0.99)	
Present study	FSS-ICU FIM		Convergent validity Correlation of 0.94 between FSS-ICU and FIM	Cronbach's alpha 0.95 Cronbach's alpha 0.86	SEM: 0.54; MDC_90%_: 1 SEM: 0.11; MDC_90%_: 1	Intrarater: ICC = 0.98 (95%CI = 0.98 - 0.99) Interrater: ICC = 0.95 (95%CI = 0.93 - 0.97) Intrarater: ICC = 0.95 (95%CI = 0.93 - 0.96) Interrater: ICC = 0.95 (95%CI = 0.93 - 0.96)	Floor: 0% Ceiling: 16% Floor: 0% Ceiling: 18%

FIM - Functional Independence Measure; FSS-ICU - Functional Status Score for the ICU; ICC - intraclass correlation coefficient; MDC90% - minimum detectable change at a 90% confidence level; SEM - standard error of the measurement; 95%CI - 95% confidence interval.

Parry et al.^(18)^ developed an observational study of 66 patients and used 4 scales for functional assessment: the FSS-ICU, Physical Function in Intensive Care Unit Test (PFIT), Perme Score, Intensive Care Unit Mobility Scale (IMS) and Short Physical Performance Battery (SPPB). The authors concluded that the FSS-ICU and PFIT had potential for and recommended its use in clinical practice and research. The authors found a minimally important change 4 to 5 times higher than that detected in our study. Most likely, the difference in agreement between the studies was due to the difference in reason for ICU admission, which was predominantly respiratory decompensation in the study by Parry et al.^(18)^ and postoperative in our study; thus, the population studied by Parry et al al.^(18)^ was potentially more clinically unstable.

Regarding interpretability, in a multicenter study, Huang et al.^(28)^ found a ceiling effect only during the assessment at hospital discharge, while our study found the same effect in the FSS-ICU during the ICU stay, which may be explained by the higher prevalence of surgical patients in our sample. In that same study,^(28)^ the internal consistency and the convergent validity were similar to those found by us. The intrarater reliability measured in our study was confirmed by the study by Ragavan et al.,^(32)^ although those authors did not follow the COSMIN^(33)^ recommendations for performing clinimetric studies.

The identification of tools to assess the limitations of activity and disability in critically ill patients who already had their measurement properties tested was the subject of a systematic review conducted by Parry et al.^(16)^ The authors found that high PFIT and FSS-ICU scores at ICU discharge are associated with discharge directly to home. However, they emphasize that it is difficult to use a single tool for physical evaluation in the ICU due to the clinical fluctuations of critically ill patients and the limitations for patient cooperation in tests, such as muscle strength assessments. The authors recommend that evaluations be based on the International Classification of Functioning, Disability and Health (ICF), which is covered by the FSS-ICU and evaluates activities (transfers and walking).^(16)^

The evaluation of the measurement properties of the FIM occurred only in hospitalized patients. Therefore, its ability to identify functional changes in the rehabilitation setting is high.^(30)^ However, the total FIM has limitations for reproduction in the ICU setting because it includes activities such as using the toilet and climbing stairs.^(34)^ Therefore, the FSS-ICU may be more appropriate for application in the ICU setting because it considers activities more common in the context of critical illness.^(34)^

The limitations of this study include the possible identification of a ceiling effect in this population due to the higher prevalence of postoperative patients in the sample, with an average length of stay of 4.5 days in the ICU, which does not allow distinguishing whether it is a limitation of the scale or of the sample size. In addition, because this was a single-center study, our results may not necessarily represent the population of critically ill patients from Brazilian ICUs; however, most studies on measurement properties have the same characteristics, and the properties found in this study were similar to those from previous studies in other languages.

## CONCLUSION

Most of the measurement properties (internal consistency, agreement, reliability, convergent validity and floor effect) of the FSS-ICU and of the FIM-motor domain are adequate for evaluating bed transfers and the walking ability of critically ill patients in the intensive care unit, although the scales have ceiling effects.
